# Special issue on bio-ontologies and phenotypes

**DOI:** 10.1186/s13326-015-0040-2

**Published:** 2015-12-17

**Authors:** Larisa N. Soldatova, Nigel Collier, Anika Oellrich, Tudor Groza, Karin Verspoor, Philippe Rocca-Serra, Michel Dumontier, Nigam H. Shah

**Affiliations:** Brunel University, London, UK; The University of Cambridge, Cambridge, UK; The Wellcome Trust Sanger Institute, Hinxton, UK; The Garvan Institute of Medical Research, Sydney, Australia; The University of Melbourne, Melbourne, Australia; The University of Oxford, Oxford e-Research Centre, Oxford, UK; Stanford University, Stanford, CA USA

## Abstract

The bio-ontologies and phenotypes special issue includes eight papers selected from the 11 papers presented at the Bio-Ontologies SIG (Special Interest Group) and the Phenotype Day at ISMB (Intelligent Systems for Molecular Biology) conference in Boston in 2014. The selected papers span a wide range of topics including the automated re-use and update of ontologies, quality assessment of ontological resources, and the systematic description of phenotype variation, driven by manual, semi- and fully automatic means.

## Introduction

The special issue on bio-ontologies and phenotypes includes selected papers that were presented at the Bio-Ontologies SIG and the Phenotype Day at ISMB (Intelligent Systems for Molecular Biology) conference in 2014.

Over the 17 years, the Bio-Ontologies SIG at ISMB has provided an environment for discussion of ontologies, their applications to biology, and more generally the organisation, presentation and dissemination of knowledge in biomedicine and the life sciences. In 2014 the bio-ontologies SIG ran an extended event called the Phenotype Day, which focused on the systematic description of phenotypic variation. The Phenotype Day brought together researchers across many disciplines to discuss phenotype-related issues and resources, and to share their experience with defining, representing, processing and using phenotype data.

The 2-day event on July 11th and 12th co-located with ISMB 2014 in Boston, received 38 submissions, including 26 papers, five flash updates and six poster abstracts and one position paper. Of the 11 papers selected for presentation at the meeting, the eight papers selected for this special issue are extended versions of seven original papers and one position paper.

## Summary of selected papers

The selected for the special issue papers span a wide range of topics including the automated re-use and update of ontologies, quality assessment of ontological resources, and the systematic description of phenotype variation, driven by manual, semi- and fully automatic means. From these articles, it is clear that systematic description of phenotypes plays a role when accessing and mining medical records as well as in the analysis of model organism data, genome sequence analysis and translation of knowledge across species. Accurate phenotyping has the potential to bridge studies that aim to advance the science of medicine, such as a better understanding of the genomic basis of diseases in the Mouse Genome Informatics (MGI) database, and studies that aim to advance the practice of medicine such as the treatment of complex disorders like Chronic Obstructive Pulmonary Disorder (COPD) [1].

Collier et al. in the paper titled “Concept selection for phenotypes and diseases using learn to rank” [2] present a supervised learning based approach for the recognition of disorder-related descriptions in electronic health records (EHRs). The authors explore the potential of four off-the-shelf concept recognition systems on the ShARE/CLEF 2013 gold standard collection and combine the systems using a variety of learn-to-rank algorithms. The four systems are Apache cTAKES, the NCBO Annotator, BeCAS and MetaMap. The proposed ensemble approach leads to an improvement in harmonized mean for recall-precision (F1 = 0.24) due primarily to an increase in recall for mentions of diseases and anatomical abnormalities. However performance across semantic types varies widely. The authors conclude with a discussion of the limitations offered by using off-the-shelf approaches and consider the need of domain adaptation in an operational setting.

In “Development and validation of a classification approach for extracting severity automatically from electronic health records” Boland et al. [3] present a method for classifying phenotype-level severity between severe and mild conditions such as Acne, which would be considered mild, compared to say heart failure. The authors report that previous machine learning approaches have tended to yield high false positive rates due to the large space of phenotypes. The random forest approach they propose exploits several measures of severity such as cost, treatment time, medications and procedure to yield a sensitivity of 0.92 and a specificity of 0.78 in discerning mild phenotypes from severe conditions when compared to a gold standard corpus.

Funk et al. in the paper titled “Evaluating a variety of text-mined features for automatic protein function prediction with GOstruct” report the results of their analysis on the use of protein-related features extracted from the biomedical literature for prediction of protein functions [4]. The authors considered two approaches: (1) a knowledge-based approach that uses ontology concepts co-mentions and is based on co-occurences of an entity and the corresponding ontology terms identified in the literature; (2) a knowledge-free approach that uses a bag-of-words as features; and where proteins are associated to words from the sentences in which they are mentioned. Two data sources were used in this study: abstracts and titles from Medline, and full-text articles from the PubMed Open Access Collection (PMCOA). Gene Ontology (GO) annotations for human and yeast genes were obtained from the GOA (Gene Ontology Annotation) datasets. The authors analysed the impact of using the two alternative approaches for feature construction on the quality of prediction of protein functions. Interestingly, both approaches provided similar levels of performance. Overall, the best performance is seen when using both co-mentions and the bag-of-words features, but the advantage is marginal. Funk et al. have demonstrated that the ability to recognize GO terms in the literature text leads to more informative functional predictions [4].

Smith et al. present a paper titled “Expanding the mammalian phenotype ontology to support automated exchange of high throughput mouse phenotyping data generated by large-scale mouse knockout screens” [5]. The authors expand and exploit the Mammalian Phenotype (MP) ontology for the annotation and organization of high throughput data from phenotype screening experiments in the MGI database. The authors discuss how recent additions and revisions have been undertaken in many areas of the MP to support automated data exchange with the International Mouse Phenotype Consortium and other projects. In total 287 new terms were added to the MP hierarchy during the present revision. The majority of the terms were added in the homeostasis/metabolism section of the ontology.

Fu et al. [6] in the paper titled “Supporting the annotation of chronic obstructive pulmonary disease (COPD) phenotypes with text mining workflows” presents a methodology for constructing a corpus of full-text clinical documents for fine-grained COPD annotations. The authors report that symptoms from COPD vary widely between patients so that automated free-text analysis on the EHR is necessary to bring pertinent conditions to the attention of the clinician. However, gold standard data for COPD related symptoms have so far been lacking, hampering the development of text mining approaches. Annotation efforts by Fu et al., were supported by expert-led guideline development and the re-use of the Argo text mining platform yielding an F1 of 0.46 using relaxed matching.

“eNanoMapper: harnessing ontologies to enable data integration for nanomaterial risk assessment” by Hastings et al. presents an ontology which covers broad areas such as a categorisation of nanoparticle classes based on their properties, constituency and shape, physicochemical and biological properties of nanoparticles, environmental aspects, experimental design, as well as safety information [7]. Following the best practices in ontology development, eNanoMapper re-uses existing ontologies, i.e. ChEBI (Chemical Entities of Biological Interest), NPO (NanoParticle Ontology), BAO (BioAssay Ontology). To overcome the limitations of the manual imports from third party ontologies, the authors developed a dedicated library to facilitate ontology re-use by extracting subsets of existing ontologies, which allows the resulting branches and components of different ontologies to be automatically pieced together.

Winnenburg et al. in the paper titled “Using description logics to evaluate the consistency of drug-class membership relations in NDF-RT” compared the asserted and inferred class relations in the recently updated NDF-RT (National Drug File Reference Terminology) ontology [8]. NDF-RT integrated authoritative drug-class membership assertions extracted from the Structured Product Labels by FDA (the Food and Drug Administration). The authors evaluated the consistency of the drug-class membership relations inferred from the pharmacologic class definitions and drug descriptions, against the newly asserted, authoritative drug-class membership relations. The enriched logic in NDF-RT enables the evaluation of the quality and completeness of newly added knowledge. The authors conclude that the inferred and asserted relations matched only in about 50 % of the cases. The results suggest that there is an opportunity for quality assurance of NDF-RT content (completeness of the drug descriptions and quality of the class definitions).

In a commentary by Papatheodorou et al. titled “Linking gene expression to phenotypes via pathway information” the authors survey recent research efforts to provide knowledge support for managing and integrating data about genes, pathways and phenotypes [9]. The authors argue that in order to exploit the full potential of gene expression data to infer phenotypic consequence due to changes in gene expression, the links between gene expression and pathways as well as pathways and phenotypes need to be improved. Furthermore, the authors suggest that the current ontological representations of phenotypes needs to be extended, in order to cover the complexity and variability of phenotypes in and across species. They summarize that the current state-of-the-art builds solid foundations for future work to overcome these challenges.

## Conclusion

In recent years the biological sciences have generated very large, complex data sets whose management, analysis and sharing have created unprecedented challenges. The development of bio-ontologies has been critical in handling these data and enabling interoperability between databases and between applications [10]. The systematic description of phenotype variation is crucial for elucidating the causal relationship between a genotype placed in a certain environment and a phenotype. Systematic representation of phenotypes using ontologies is essential when accessing and mining medical records as well as for the analysis of model organism data, genome sequence analysis and translation of knowledge across species. The papers included in the bio-ontologies and phenotypes special issue report on the development, management and quality assessment of ontological resources, and the systematic description of phenotype variation, driven by manual, semi- and fully automatic means.
